# Corneal Culture in Infectious Keratitis: Effect of the Inoculation Method and Media on the Corneal Culture Outcome

**DOI:** 10.3390/jcm10091810

**Published:** 2021-04-21

**Authors:** Susanna Sagerfors, Chrysoula Karakoida, Martin Sundqvist, Birgitta Ejdervik Lindblad, Bo Söderquist

**Affiliations:** 1 Department of Ophthalmology, Faculty of Medicine and Health, Örebro University, SE 701 82 Örebro, Sweden; birgitta.ejdervik-lindblad@regionorebrolan.se; 2 Department of Laboratory Medicine, Clinical Microbiology, Faculty of Medicine and Health, Örebro University, SE 701 82 Örebro, Sweden; xrkarakoida@hotmail.com (C.K.); martin.sundqvist@regionorebrolan.se (M.S.); bo.soderquist@oru.se (B.S.)

**Keywords:** infectious keratitis, corneal culture, indirect inoculation, direct inoculation

## Abstract

Background: To compare two different methods of corneal culture in infectious keratitis: multiple sampling for direct inoculation and enrichment (standard method) and a single sample via transport medium for indirect inoculation (indirect inoculation method). Methods: Prospective inclusion of patients fulfilling predefined criteria of infectious keratitis undergoing corneal culture according to both studied methods in a randomized order. Results: The standard method resulted in a significantly higher proportion of positive culture outcomes among the 94 included episodes of infectious keratitis (61%; 57/94) than the indirect inoculation method (44%; 41/94) (*p* = 0.002) and a significantly higher proportion of microorganisms than the indirect inoculation method, with a Cohen’s kappa of 0.38 (95% CI: 0.28–0.49) for agreement between the methods. Subanalysis of culture results showed that direct inoculation on gonococcal agar only combined with the indirect inoculation method resulted in a similar rate of culture positive patients and proportion of detected microorganisms to the standard method. Conclusion: Indirect inoculation of one corneal sample cannot replace direct inoculation of multiple corneal samples without loss of information. A combination of directly and indirectly inoculated samples can reduce the number of corneal samples by four without statistically significant differences in culture outcome or in the proportion of detected microorganisms.

## 1. Introduction

Infectious keratitis, caused by bacteria, fungi, or protozoa, is a common ophthalmological emergency that can lead to permanent loss of vision. Geographical variations are reported in both the microbial spectrum [1] and the antibiotic susceptibility pattern [2,3]. Identification of the disease-causing pathogen(s) allows individualized treatment that may reduce the risk of emergence of reduced susceptibility to antibiotics. It also forms the basis of empirically guided therapy recommendations before corneal culture reports are available [4]. The diagnostic procedure for infectious keratitis suggested by Jones et al. in 1981 was to prepare smears for staining and to perform direct inoculation on solid media (blood, chocolate, Sabouraud, and anaerobic agar plates) and inoculation in liquid media (supplemented thioglycolate broth and brain–heart infusion (BHI) broth) [5] This meticulous approach advocates at least 16 samples from the corneal infiltrate. Since then, surveys among ophthalmologists have revealed that 13–49% [6,7] treat infectious keratitis without first performing corneal culture and that 76–78% [6,7] of surveyed ophthalmologists lacked access to culture media. More recently, from Toronto, Tam et al. reported a significant decrease in the number of performed corneal scrapings from 2000 to 2015 [8].

Globally, infectious keratitis is estimated to cause unilateral blindness in 1.5–2 million cases per year [9] and infectious keratitis is estimated to cost USD 245 million in the US alone [10]. Today, a method for the detection of disease-causing pathogen(s) with no false positive or negative results is still lacking. The different components of the procedure of corneal culture in infectious keratitis have been studied previously, such as the antimicrobial effect of the topical anaesthetics used [11,12,13] or which solid culture media [14,15], or sampling instrument to use [16,17,18,19] and whether to inoculate the corneal sample directly on culture media or indirectly [20,21]. The culture media used for corneal culture in infectious keratitis have a short shelf life, and need to be stored in a refrigerator. The procedure is time consuming, and can be experienced as distressing for the patient, therefore, streamlining of the corneal culture procedure is needed. With variations in both the microbial spectrum [1] and the culture positive rate [10], new methods of detecting disease-causing pathogen(s) need to be studied and evaluated in different settings and climates.

The primary aim of this prospective study was to compare two different methods of corneal culture in infectious keratitis: our standard method, i.e., multiple sampling from the cornea using cotton-tipped applicators and knife blades directly inoculated on solid media and one sampling using a knife blade dispensed in liquid enrichment media, versus a single sample obtained using a nylon-tipped swab dispensed in a transport medium for subsequent inoculation on culture media at the laboratory (indirect inoculation). We also aimed to study if our standard method could be simplified in terms of the culture media used without loss of information, and if indirect inoculation provides additional information.

## 2. Materials and Methods

### 2.1. Study Population

This prospective study consecutively included all patients aged 18 or over presenting with suspected infectious keratitis (i.e., a corneal infiltrate with an overlying epithelial defect) at the Department of Ophthalmology, Örebro University Hospital, Sweden from 10 September 2018 to 27 January 2020. Patients were included if either or both of the following were true: (1) the corneal culture from the infiltrate showed growth of bacteria, fungi, or protozoa (any microbial growth was regarded as significant), (2) the patient fulfilled the clinical criteria of infectious keratitis, that is, corneal infiltrate with overlying epithelial defect in combination with at least one of the following: infiltrate within/overlapping the central 4 mm of the cornea and/or uveitis and/or pain [22]. The exclusion criterion was corneal culture not performed according to study protocol.

Patients completed a questionnaire regarding symptom duration, general health, previous eye diseases or surgeries, and contact lens habits (if applicable). The ophthalmologist or resident in ophthalmology documented the patient’s clinical status, predisposing risk factors for keratitis, and whether topical antibiotic treatment had preceded the corneal culture, and then performed corneal cultures according to both the standard method and the indirect inoculation method in a randomized sampling order. Information on age, best corrected visual acuity (BCVA) in Snellen decimals, and treatment was collected from the medical charts.

### 2.2. Corneal Sampling

**Figure 1:**
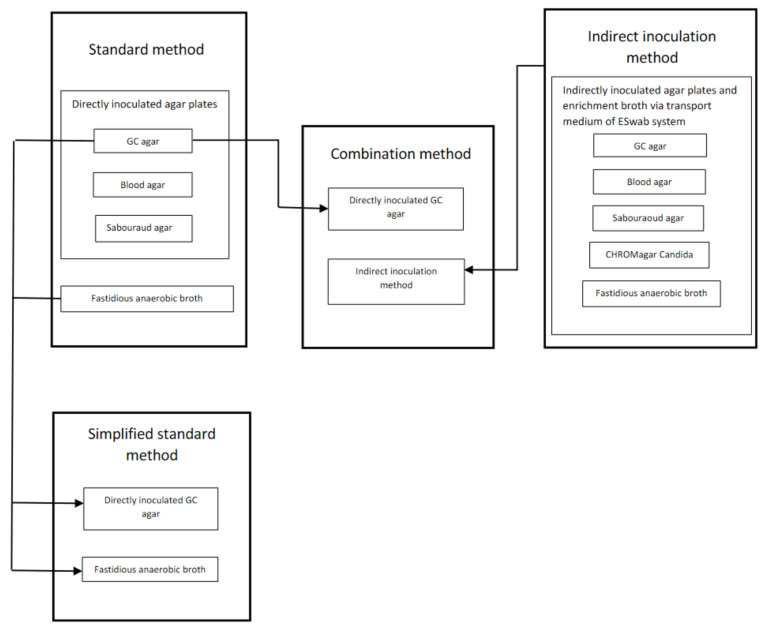
Direct and indirect inoculation approaches and culture media used in four different methods of corneal culture in patients with infectious keratitis.

The cornea was anesthetized with topical non-preserved tetracaine. The standard method of corneal culture at our ophthalmological department, a tertiary referral corneal center, was in accordance with the Swedish State of the Art Document “Infectious Keratitis Caused by Bacteria, Fungi, and Protozoa” during the study period, that is, corneal samples (*n* = 7) obtained using both sterile cotton-tipped applicators (*n* = 3) and knife blades (*n* = 3) that were directly inoculated onto gonococcal (GC), blood and Sabouraoud (SAB) agar plates. Finally, a corneal sample was obtained using a knife blade (*n* = 1) that was dispensed in fastidious anaerobic broth (FAB) for enrichment and for anaerobe inoculation (Figure 1). The indirect inoculation method included one single corneal sample from the infiltrate obtained using the nylon-tipped swab from the ESwab system (COPAN Italia S.p.A., Brescia, Italy) that was dispensed in the liquid Amies medium of the same system for indirect inoculation on agar plates at the laboratory (see Appendix A).

Each patient was sampled and cultured according to both methods, the standard method and the indirect inoculation method, and we chose to apply a similar approach asPakzad-Vaezi et al. [23]. In those cases where the standard method was randomized as “standard method first”, the sample for the indirect inoculation method was performed after the direct inoculation on the GC agar plate. This meant that patients randomized to the sampling order “standard method first” initially had corneal samples taken for direct inoculation on a GC agar plate, then a corneal sample for indirect inoculation with the nylon-tipped swab that was dispensed in the transport medium of the ESwab system (COPAN Italia S.p.A., Brescia, Italy). Patients randomized to “indirect inoculation first” were initially sampled according to the indirect inoculation method with the swab and transport medium provided by the ESwab system, then sampled for direct inoculation on a GC agar plate. The sampling sequences for the remaining media were then equal for “standard method first” and “indirect inoculation first”, namely, samples were collected for direct inoculation on blood agar and SAB agar and a final sample was dispensed in FAB.

For each medium used, the cornea was repeatedly sampled with a new instrument. All media were incubated within 24 h. If incubation was not performed in connection with the sampling, the media were stored in a refrigerator at +5 °C. For detailed information regarding incubation conditions, contents of the culture media used, and handling of the sample in transport media, see (Appendix A).

Regarding the pathogenic potential of the isolated bacteria, we applied the definition by Fleiszig and Efron of classifying coagulase-negative staphylococci (CoNS), *Corynebacterium* spp. (except *Corynebacterium diphtheriae)*, *Cutibacterium acnes*, *Micrococcus* spp., *Bacillus* spp., and *Peptostreptococcus* spp. as normal flora. However, we chose the term “commensals” for the purpose of this study instead of “normal flora” since the microorganisms were recovered from corneal ulcers. All other bacteria were classified as “potentially pathogenic” [24]. We chose to classify fungal species as “potentially pathogenic”.

### 2.3. Statistical Analysis

Power calculation was performed by a statistician according to the primary aim of the study, to compare the standard culture method with the indirect inoculation method, and was based on the results from the study by Pakzad-Vaezi et al. [23] with an agreement on culture results between the two investigated culture methods in 85% of the patients. This gave a sample size of 100 patients. The corneal sampling order of “standard method first” or “indirect inoculation first” was randomized by a statistician by block randomization using version 25 of the IBM SPSS software package. Envelopes numbered from 1 to 110 with the randomized sampling order, that is, either “standard method first” or “indirect inoculation first”, were prepared by a secretary not involved with the care of patients with infectious keratitis; the reason for preparing 110 envelopes was that we assumed an exclusion rate of approximately 10%. After obtaining written consent from the patient, the envelope with the lowest number was opened and corneal culture was performed according to the randomized sampling order and instructions inside.

Comparisons on a group level (i.e., of the proportion of culture positive patients with infectious keratitis in relation to culture method) were calculated in version 25 of the IBM SPSS software package using McNemar’s test. A significance level of 0.05 was chosen for all comparisons. Agreement between the different culture methods on a microorganism level was calculated as Cohen’s kappa, and as positive and negative agreement, with 95% confidence intervals (CIs). Proportions of isolated microorganisms were compared using McNemar’s test. These statistical analyses were performed through the Vassarstat.net website [25]. For statistical calculations on all isolated microorganisms and subgroups after pathogenicity and by lesion size at the time of culture, the culture negative readings by both investigated methods were summed up as described by Pakzad-Vaezi et al. [23].

## 3. Results

### 3.1. Study Cohort

During the study period, 110 episodes of infectious keratitis in 105 patients were included. One patient who was not yet 18 years old was initially considered for inclusion twice, but excluded due to not fulfilling the inclusion criterion concerning age. Another 14 patients were excluded, 13 because of cultures not performed according to study protocol and one due to lack of written consent. The final study cohort consisted of 94 episodes of infectious keratitis in 90 patients (four patients had two episodes each). Median age at episode onset was 44 years (range 18–84 years), 56% (*n* = 53) were men, 52% (*n* = 49) were randomized to sampling order “standard method first” and the right eye was affected in 52% (*n* = 49) of the episodes. The most common risk factor for infectious keratitis was contact lens wear (71%, *n* = 67). Median visual acuity in Snellen equivalents at the time of corneal culture was 0.9 (range: 0.001–1.0) (Table 1).

### 3.2. Comparison between a Multiple Sampling Method on Solid Media and Liquid Enrichment Media (Standard Method) and a Single Sampling Method Dispensed in Transport Media for Indirect Inoculation (Indirect Inoculation Method)

In total, 66% of the episodes of infectious keratitis (62/94) displayed a positive corneal culture with either or both of the investigated corneal culture methods; that is, the standard method and/or the indirect inoculation method. On the group level, the standard method gave rise to a higher rate of culture positive episodes (61%; 57/94) than the indirect inoculation method (44%; 41/94) (*p* = 0.002). On the patient level, 49% (46/94) of the patients had consistent culture results between the standard method and the indirect inoculation method; that is, a positive outcome with identical bacterial or fungal species isolated (15%; 14/94) or a negative outcome (34%; 32/94). In 38% (36/94) of the patients, the culture results were inconsistent, with the two methods either disagreeing on culture outcome (28%; 26/94), or if agreeing on a positive corneal culture, disagreeing on the bacterial or fungal species isolated (11%; 10/94). Among the remaining 13% (12/94) of the patients, the culture results between the two methods partially agreed; that is, they agreed on at least one bacterial/fungal species isolated in cultures displaying polymicrobial growth.

A total of 117 isolates, both bacterial (*n* = 116) and fungal (*n* = 1), were detected from the 62 patients with a positive corneal culture outcome. The most frequently detected microorganism was CoNS (*n* = 41). Of the detected microorganisms, 26% (30/117) were identified by both methods, 50% (59/117) by the standard method only, and 24% (28/117) by the indirect inoculation method only. The agreement on the microbial level between the standard method and the indirect inoculation method in terms of Cohen’s kappa was 0.38 (95% CI: 0.28–0.49) (Supplementary Table S1). The standard method detected a significantly higher proportion of microorganisms in total than the indirect inoculation method, but there was no significant difference for the subgroup of microorganisms that were classified as potentially pathogenic (Table 2).

Subanalysis of the microorganisms recovered by a direct inoculation approach on solid media only (Supplementary Table S1) (i.e., samples obtained using cotton-tipped applicators and knife blades directly inoculated onto GC, blood, and SAB agar plates) with those recovered by an indirect approach using the nylon-tipped swab and the liquid Amies transport medium of the ESwab system subcultured on the same plates including a CHROMagar Candida plate (Figure 1), Cohen’s kappa for all detected microorganisms was 0.49 (95% CI: 0.37–0.61). The direct inoculation approach detected a significantly higher proportion of bacteria and fungi (4%) than the indirect approach (3%) (*p* = 0.004601).

Of the 15 patients who had received topical antibiotic treatment prior to corneal culture, six displayed positive corneal cultures with a total of eight microorganisms. One of these, *Corynebacterium propinquum,* was detected by both direct inoculation on solid media and by indirect inoculation via transport medium. The remaining seven microorganisms were isolated by an indirect approach through the liquid media used; that is, by enrichment in FAB, by the transport medium, or both. No patient had a positive corneal culture by direct inoculation on solid media only. Inoculation via liquid media detected a significantly higher rate of microorganisms among the 15 patients (8/15) than direct inoculation on solid media (1/15) (*p* = 0.015625). The rate of culture positive patients was 6/15 by the liquid media used, compared to a patient positivity rate of 1/15 by direct inoculation on solid media, among patients treated with antibiotics prior to culture. This difference was not statistically significant (*p* = 0.0625).

### 3.3. Evaluation of a Simplified Standard Method and a Combination Method of Corneal Culture

Based on the existing culture results from the standard method and the indirect inoculation method, we evaluated two modified methods of corneal culture (Figure 1): a simplified standard method including only culture results from the directly inoculated GC agar and the FAB, and a combination method consisting of the culture results from the directly inoculated GC agar plate and the indirect inoculation method. The GC agar plate was chosen since most microorganisms, even the most fastidious, can be cultured on this medium. At a group level, there were no statistically significant differences in the rate of culture positive patients between the standard method (61%; 57/94) and either the simplified standard method (55%; 52/94) or the combination method (56%; 53/94).

On the patient level, consistency between the standard method and the simplified standard method was 88.3% (83/94), while that between the standard method and the combination method was 57.4% (54/94). In terms of positive outcomes, the simplified and combination methods detected bacterial or fungal species identical to those detected by the standard method in 48.9% (46/94) and 23.4% (22/94) of episodes, respectively. The corresponding figures regarding negative outcomes were 39.4% (37/94) for the simplified standard method and 34.0% (32/94) for the combination method. Disagreements on culture outcome were noted in 5.3% (5/94) and 14.9% (14/94) of episodes, respectively, and disagreements regarding detected species were noted in 0% and 7.4% (7/94), respectively. In the remaining 6.4% (6/94) and 20.2% (19/94) of the episodes, the culture results partially agreed on the identity of at least one isolated microorganism in samples displaying polymicrobial growth.

On the microbial level, the simplified standard method resulted in a statistically significantly lower proportion of detected microorganisms (*p* = 0.000488) compared to the standard method (Table 3). Of the 12 microorganisms that were not detected by the simplified standard method, only one, *Staphylococcus aureus,* was considered potentially pathogenic. The remaining 11 microorganisms were *Staphylococcus epidermidis* (*n* = 7), other CoNS (*n* = 1), and *C. acnes* (*n* = 3). Cohen’s kappa agreement between the standard method and the simplified standard method was 0.92 (95% CI: 0.88–0.97) (Table 3). 

There were no statistically significant differences in the proportion of detected microorganisms between the standard method and the combination method, regardless of presumed pathogenicity (Table 3). Cohen’s kappa agreement between the standard method and the combination method was 0.63 (95% CI: 0.54–0.74) (Table 3).

## 4. Discussion

In this prospective study of patients with infectious keratitis, we compared our standard corneal culture method, which included seven samplings using cotton-tipped applicators and knife blades from the ulcer directly inoculated on agar plates and in liquid enrichment medium, with a “single sample” technique using a nylon-tipped swab dispensed in a transport medium

On the microbial level, we could only demonstrate a “fair” agreement according to the interpretations of Cohen’s kappa by Landis [26] and Altman [27] between the standard method of corneal culture and the single sample technique with the indirect inoculation method in patients with infectious keratitis. The standard method resulted in a significantly higher proportion of detected microorganisms than the indirect inoculation method, but there was no significant difference in the detected proportion of the subgroup of microorganisms considered potentially pathogenic.

Our findings are similar to those reported by Nielsen et al. [28]. In the Corneal Ulcer One-Touch Study, Pakzad-Vaezi et al. reported greater agreement on the microbial level than in our study [23]. One reason for this may be a slight difference in inclusion criteria. However, we could not confirm the same level of agreement as Pakzad-Vaezi et al. after subgrouping our study cohort according to lesion size. The difference in the distribution of underlying risk factors could be an explanation or differences in the spectrum of pathogens or different sampling instruments of corneal material for direct inoculation.

The most commonly isolated microorganisms in our study were CoNS, and this finding of bacteria that are considered to constitute the normal flora of the ocular surface conforms with other reports on infectious keratitis from a similar climate [29,30].

The inclusion of a liquid medium in the corneal culture method may be advantageous if the patient has been treated with topical antibiotics prior to corneal culture [31,32]. In the present study, we did observe a statistically significantly higher proportion of detected microorganisms among the 15 patients treated with antibiotics prior to sampling for culture when we used an indirect approach of dispensing the sample in a liquid medium prior to plating on solid agar, compared to direct inoculation on solid agar. The difference in the proportion of culture positive patients was not significant, but we cannot exclude the possibility that this lack of significance was due to a small sample size.

The standard method involves seven samplings for inoculation on three different agar plates and in FAB. Reducing the number of plates to one, the GC agar, the method could be simplified and the number of samplings reduced to three. If applied on existing culture results, this simplified standard method resulted in the loss of 12 microorganisms, of which only one (*S. aureus*) was an indisputable corneal pathogen. This is similar to the findings by Waxman et al. and Das et al. [14,15].

We also studied the effect of combining culture results from the directly inoculated GC agar plates with the culture results from the indirect inoculation method. The agreement on the microbial level between this combination method and the standard method was less than the agreement between the simplified standard method and the standard method, but could still be considered “substantial” according to the definition by Landis [26] or “good” according to the definition by Altman [27]. In addition, the proportion of microorganisms detected by the combination method was not statistically different from the proportion detected by the standard method irrespective of presumed pathogenicity.

This study has some potential limitations. The microbiologists reading the agar plates were not blinded, however, any microbial growth was regarded as significant and further analyzed by MALDI-TOF MS. The samples were obtained using cotton-tipped applicators and knife blades according to the standard method, and by a nylon-tipped swab according to the indirect inoculation method. The influence of the sampling instrument on culture outcome and culture results was not aimed to be investigated in this present study and applications and conclusions drawn from this study must therefore take this into account, which may be considered as a limitation. Fungal ulcers are rare in our part of the world, and so the present results are not necessarily transferable to a context where fungal ulcers are more common. The present study included all cases presenting with suspected infectious keratitis regardless of lesion size or disease severity, which may be considered a limitation due to possible difficulties in obtaining a representative culture sample from small ulcers. This may also be considered a strength, since this cohort represents the cases presenting with suspected infectious keratitis in our region. Another strength of the study is the prospective design with a randomized sampling order, with a study period extending for more than a year and hence including all seasons. A final strength is our choice to evaluate culture media and methods for corneal culture on multiple levels: the group, patient, and microbial levels.

In conclusion, in a clinical context of bacterial ulcers of limited size and severity, multiple sampling for direct inoculation on agar plates and enrichment in FAB using cotton-tipped applicators and knife blades gives rise to a higher rate of culture positivity and a higher proportion of isolated microorganisms than a single sample using a nylon-tipped swab dispensed in liquid Amies medium for indirect inoculation. Direct inoculation on GC agar plates combined with indirect inoculation reduces the number of samplings without any significant information loss compared to multiple sampling.

## Figures and Tables

**Table 1:** Background data on the study cohort treated for infectious keratitis (*n* = 94 episodes).

Age in years, median (range)	44.0 (18–84)	
Male sex, *n* (%)	53 (56.4%)	
Right eye, *n* (%)	49 (52%)	
Days with symptoms prior to culture ^a^, median (range)	3.0 (0.5–61)	
Largest diameter ^b^ (mm), median (range)	1.0 (0.1–8.0)	
Lesion area ^b^ (mm^2^), median (range)	0.8 (0.01–50.2)	
Topical antibiotic prior to corneal culture, *n* (%)	15 (16%)	
Risk factors for keratitis	Contact lens wear, *n* (%)	67 (71.3%)
	Ocular surface disease, *n* (%)	13 (13.8%)
	No identified risk factor, *n* (%)	7 (7.4%)
	Prior ocular surgery, *n* (%)	4 (4.3%)
	Trauma, *n* (%)	3 (3.2%)
BCVA ^c^ at time of corneal culture, median (range)	0.9 (0.001–1.0)	

^a^ Information on the duration of symptoms was missing for three patients. ^b^ Information on lesion size was missing for one patient. ^c^ BCVA: best corrected visual acuity, Snellen decimals. Information on visual acuity at the time of corneal culture was missing for six patients, and one patient was amaurotic.

**Table 2:** Agreement and differences between the standard corneal culture method and the indirect inoculation method for microorganisms isolated from infectious keratitis.

						Agreement, Cohen’s Kappa (95% CI)			Method Difference		*p*-Value ^f^
H		Number of microorganisms isolated by standard method and indirect inoculation method	Number of microorganisms isolated by standard method only	Number of microorganisms isolated by indirect inoculation method only	Number of culture negative readings by both standard method and indirect inoculation method	Agreement between standard method and indirect inoculation method	Positive agreement between standard method and indirect inoculation method	Negative agreement between standard method and indirect inoculation method	Proportion of culture positive readings using standard method	Proportion of culture positive readings using indirect inoculation method	
	Total	30	59	28	1669	0.38 (0.28–0.49)	0.41 (0.30–0.52)	0.97 (0.97–0.98)	0.0498	0.0325	0.001169
Comparison of detected microorganisms according to lesion size ^a^	Large (largest diameter ≥1.0 mm) ^b^	16	43	20	1595	0.32 (0.19–0.44)	0.33 (0.22–0.47)	0.98 (0.97–0.99)	0.0352	0.0215	0.005152
	Small (largest diameter ≤0.9 mm) ^c^	12	15	8	709	0.50 (0.32–0.67)	0.5 (0.31–0.69)	0.98 (0.97–0.99)	0.0363	0.0269	0.21004
Comparison of detected microorganisms according to presumed pathogenicity	Potentially pathogenic ^d^	7	11	4	1200	0.48 (0.25–0.70)	0.47 (0.25–0.70)	0.99 (0.99–1.0)	0.0147	0.009	0.118469
	Commensals ^e^	23	48	24	469	0.32 (0.20–0.44)	0.39 (0.28–0.52)	0.93 (0.90–0.95)	0.1259	0.0833	0.00631

^a^ One patient excluded due to missing information on lesion size. ^b^ Sixty episodes had a large diameter of ≥1.0 mm. ^c^ Thirty-three episodes had a small diameter of ≤0.9 mm. ^d^ Potentially pathogenic [24]: *Staphylococcus aureus*, *Streptococcus pneumoniae*, *Enterococcus faecalis*, *Brachybacterium* spp., *Streptococcus mitis*, *Enterobacter cloacae*, *Pantoea* spp., *Pseudomonas aeruginosa*, *Moraxella* spp., *Haemophilus parainfluenzae*, *Cutibacterium avidum*, *Veillonella parvula*, *Candida albicans*. ^e^ Commensals [24]: coagulase-negative staphylococci (CoNS), *Corynebacterium* spp., *Cutibacterium acnes*, *Micrococcus* spp. ^f^ McNemar’s test.

**Table 3:** Agreement and differences on a microbial level between the standard corneal culture method and two modified methods, a simplified standard method and a combination method, in patients with infectious keratitis.

					Agreement, Cohen’s Kappa (95% CI)			Method Difference		*p*-Value ^c^
	Number of microorganisms isolated by both methods: standard method and either simplified standard method or combination method	Number of microorganisms isolated by standard method only	Number of microorganisms isolated by either simplified standard method only or combination method only	Number of culture negative readings from both methods: standard method and either simplified standard method or combination method	Agreement between standard method and either simplified standard method or combination method	Positive agreement between standard method and either simplified standard method or combination method	Negative agreement between standard method and either simplified standard method or combination method	Proportion of microorganisms detected by standard method	Proportion of microorganisms detected by either simplified standard method or combination method	
Simplified standard method ^a^										
Total	77	12	0	1415	0.92 (0.88–0.97)	0.93 (0.85–0.97)	1.0 (0.99–1.0)	0.0592	0.0512	0.00049
Potentially pathogenic ^d^	17	1	0	1016	0.97 (0.91–1)	0.94 (0.74–0.99)	1.0 (0.99–1.0)	0.0174	0.0164	1.00
Commensals ^e^	60	11	0	399	0.90 (0.85–0.96)	0.91 (0.82–0.96)	0.99 (0.97–0.99)	0.1511	0.1277	0.00977
Combination method ^b^										
Total	56	33	28	1669	0.63 (0.54–0.72)	0.64 (0.54–0.74)	0.98 (0.97–0.99)	0.0498	0.047	0.60892
Potentially pathogenic ^d^	13	5	4	1200	0.74 (0.57–0.90)	0.72 (0.49–0.88)	1.0 (0.99–1.0)	0.0147	0.0139	1.00
Commensals ^e^	42	28	24	469	0.57 (0.47–0.68)	0.62 (0.51–0.73)	0.95 (0.92–0.96)	0.1259	0.1188	0.67781

^a^ Simplified standard method: direct inoculation on GC agar plates and enrichment via fastidious anaerobic broth. ^b^ Combination method: direct inoculation on GC agar plates from standard method and indirect inoculation method. ^c^ McNemar’s test. ^d^ Potentially pathogenic [24]: *Staphylococcus aureus, Streptococcus pneumoniae, Enterococcus faecalis, Brachybacterium* spp., *Streptococcus mitis, Enterobacter cloacae, Pantoea* spp., *Pseudomonas aeruginosa, Moraxella* spp., *Haemophilus parainfluenzae, Cutibacterium avidum, Veillonella parvula*, *Candida albicans*. ^e^ Commensals [24]: coagulase-negative staphylococci (CoNS), *Corynebacterium* spp., *Cutibacterium acnes*, *Micrococcus* spp.

## Data Availability

Data are contained within the article or Supplementary Material.

## Supplementary Materials

The following are available online at https://www.mdpi.com/article/10.3390/jcm10091810/s1: Table S1: Microorganisms (*n* = 117) isolated from corneal cultures of episodes of infectious keratitis (*n* = 94) according to different culture methods.

## Appendix A

### Incubation Conditions for Culture Media, the Handling of the Sample in Transport Media and Contents of the Agar Plates

The directly inoculated agar plates and the FAB were handled as follows: the GC agar plate was incubated in air with 5% carbon dioxide (CO_2_) at 36 °C, the blood agar plate was incubated at 36 °C in air the first day and in CO_2_ on the following days, and the SAB plate was incubated in air at 30 °C. All plates were examined daily for 7 days, and discarded if no growth was detected. The FAB was incubated in air at 36 °C for 7 days (or shorter if growth was detected), and thereafter subcultured on GC agar incubated in CO_2_ for 2 days and fastidious anaerobe agar (FAA; NEOGEN, Lansing, MI, USA) supplemented with 5% horse blood incubated under anaerobic conditions for 5 days.

The sample in transport medium for indirect inoculation was handled as follows. After homogenizing the transport media for 5 s by vortex, 30 µL of the suspension was inoculated on: GC agar, blood agar, SAB agar, and CHROMagar Candida (CHROMagar, Paris, France). The GC, blood, and SAB agar plates were incubated and examined as described for the directly incubated plates, and the CHROMagar Candida plate was incubated in air at 37 °C for 3 days. Thirty µL of the suspension was incubated in FAB and subcultured once growth was visible, or after 7 days if no growth was detected, on GC agar and FAA and handled as described for the standard method above.

Bacteria and fungi were identified to species or genus level using matrix-assisted laser desorption/ionization time-of-flight mass spectrometry (MALDI-TOF MS) (Microflex LT and Biotyper 3.1, Bruker Daltonik, Bremen, Germany).

GC agar plate (GC Medium Base, Becton Dickinson, Sparks, MD, USA, supplemented with chocolatized defibrinated horse blood).

Blood agar plate (3.9% Columbia Blood Agar Base, Oxoid, Basingstoke, Hampshire, UK, supplemented with 6% defibrinated horse blood).

Sabouraud (SAB) agar plate (1.3% Agar No 2, Lab M, Heywood, Bury, UK; 4% D-Glucose, VWR, Leuven, Belgium; 1% Peptone, Becton Dickinson).

Fastidious anaerobic broth (FAB) (2.97% FAB, Lab M, supplemented with 1% D-glucose, VWR).
