# Supplementary material for: Corneal Culture in Infectious Keratitis: Effect of the Inoculation Method and Media on the Corneal Culture Outcome
Source: J Clin Med. 2021 Apr 21;10(9):1810. doi: 10.3390/jcm10091810 (PMC8122416; doi:10.3390/jcm10091810)
Supplement: Supplementary file 1 [file jcm-10-01810-s001.zip › jcm-1160515-supplementary.pdf]

Supplementary material

Supplementary Table S1. Microorganisms (*n* = 117) isolated from corneal cultures of episodes of infectious keratitis (*n* = 94) according to different culture methods.

	**Number of microorganisms isolated by standard method and indirect inoculation method (number of these isolated without enrichment in FAB)**	**Number of microorganisms isolated by standard method only (number of these isolated without enrichment in FAB)**	**Number of microorganisms isolated by indirect inoculation method only (number of these isolated without enrichment in FAB)**	**Number of culture negative readings by both standard method and indirect inoculation method (number found negative without enrichment in FAB)**	**Cohen's kappa [95% CI] for agreement between standard method and indirect inoculation method (agreement between both methods without enrichment in FAB)**
Staphylococcus aureus (*n* = 5)	0 (0)	5 (5)	0 (0)	89 (89)	0 [nc[a]]<br>(0 [nc[a]])
Staphylococcus epidermidis (*n* = 31)	6 (5)	18 (10)	7 (5)	63 (74)	0.18 [0–0.40]<br>(0.31 [0.05–0.58])
Other coagulase-negative staphylococci (*n* = 10)	1 (1)	5 (4)	4 (4)	84 (85)	0.13 [0–0.45]<br>(0.16 [0–0.50])
Streptococcus pneumoniae (*n* = 1)	1 (1)	0 (0)	0 (0)	93 (93)	1 [1–1]<br>(1 [1–1])
Enterococcus faecalis (*n* = 2)	1 (1)	1 (1)	0 (0)	92 (92)	0.66 [0.04–1]<br>(0.66 [0.04–1])
Corynebacterium spp. (*n* = 24)	12 (12)	10 (10)	2 (2)	70 (70)	0.59 [0.39–0.80]<br>(0.59 [0.39–0.80])
Brachybacterium spp. (*n* = 1)	0 (0)	1 (0)	0 (0)	93	0 [0–0]
Streptococcus mitis (*n* = 1)	1 (1)	0 (0)	0 (0)	93 (93)	1 [1–1]<br>(1 [1–1])
Micrococcus spp. (*n* = 2)	0 (0)	2 (2)	0 (0)	92 (92)	0
Enterobacter cloacae (*n* = 1)	0 (0)	0 (0)	1 (1)	93 (93)	0 [0–0]<br>(0 [0–0])
Pantoea spp. (*n* = 2)	0 (0)	1 (0)	1 (1)	92 (93)	[b]<br>(0 [0–0])
Pseudomonas aeruginosa (*n* = 3)	2 (2)	1 (1)	0 (0)	91 (91)	0.79 [0.39–1]<br>(0.79 (0.40-1))
Moraxella spp. (*n* = 2)	1 (1)	0 (0)	1 (1)	92 (92)	0.66 [0.04–1]<br>(0.66 [0.04–1])
Haemophilus parainfluenzae (*n* = 1)	1 (1)	0 (0)	0 (0)	93 (93)	1 [1–1]<br>(1 [1–1])
Cutibacterium acnes (*n* = 27)	4 (1)	13 (3)	10 (0)	67 (90)	0.11 [0–0.34]<br>0.39 [0–0.93])
Cutibacterium *avidum* (*n* = 1)	0 (0)	0 (0)	1 (0)	93	0 [0–0]
Staphylococcus saccharolyticus (*n* = 1)	0 (0)	0 (0)	1 (1)	93 (93)	0 [0–0]<br>(0 [0–0])
Veillonella parvula (*n* = 1)	0 (0)	1 (0)	0 (0)	93	0 [0–0]
Candida albicans (*n* = 1)	0 (0)	1 (0)	0 (0)	93	0 [0–0]
Total (*n* = 117)	30 (26)	59 (36)	28 (15)	1669 (1333)	0.38 [0.28–0.49]<br>(0.49 [0.37–0.61])
Potentially pathogenic[c] (*n* = 22)	7 (7)	11 (7)	4 (3)	1200 (829)	0.48 [0.25–0.70]<br>(0.58 [0.34–0.81])
Commensals[d] (*n* = 95)	23 (19)	48 (29)	24 (12)	469 (504)	0.32 [0.20–0.44]<br>(0.44 [0.30–0.59])

[a] nc: quantity could not be calculated. [b] Kappa not calculated because observed concordance was smaller than mean chance. [c] Potentially pathogenic[1]: *Staphylococcus aureus, Streptococcus pneumoniae, Enterococcus faecalis, Brachybacterium* spp., *Streptococcus mitis, Enterobacter cloacae, Pantoea* spp., *Pseudomonas aeruginosa, Moraxella* spp., *Haemophilus parainfluenzae, Cutibacteriu*m *avidum, Veillonella parvula, Candida albicans*. [d] Commensals[1]: Coagulase-negative staphylococci (CoNS), *Corynebacterium* spp., *Cutibacterium acnes, Micrococcus* spp.

Reference

1. Fleiszig SM, Efron N. Microbial flora in eyes of current and former contact lens wearers. *J Clin Microbiol* **1992**; *30*:1156-1161.
